# Peroral cholangioscopy-guided transpapillary gallbladder drainage and cholecystolithotomy in the treatment of acute cholecystitis and cholelithiasis

**DOI:** 10.1055/a-2067-4636

**Published:** 2023-05-04

**Authors:** Xiao-gang Liu, Xin-yu Huang, Rui Huang, Ren-yi Zhang, Wei-hui Liu

**Affiliations:** Department of Gastroenterology and Hepatology, Sichuan Academy of Medical Sciences & Sichuan Provincial Peopleʼs Hospital, School of Medicine, University of Electronic Science and Technology of China, Chengdu, Sichuan Province, China

A 35-year-old pregnant woman presented with upper right abdominal pain and jaundice. Laboratory analysis showed leukocytosis, elevated C-reactive protein, hypertransaminasemia, and cholestasis. Endoscopic ultrasonography (EUS) revealed cholecystolithiasis, acute cholecystitis, and suspected choledocholithiasis ( Fig. 1 ). The patient was referred for endoscopic retrograde cholangiopancreatography (ERCP) treatment for gallbladder drainage and choledocholithiasis removal ( Video 1 ).

**Fig. 1:**
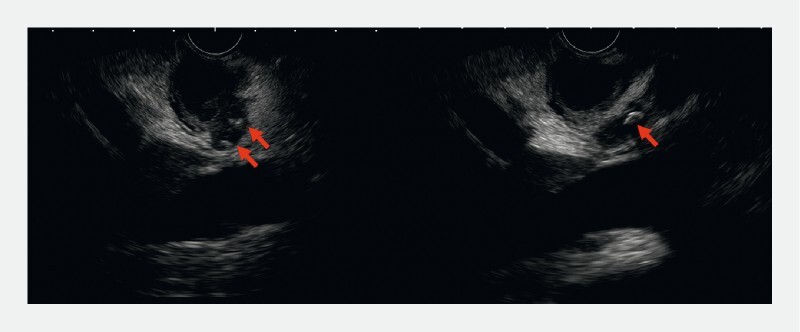
Endoscopic ultrasonography images showing a distended gallbladder, cholecystolithiasis, and suspected choledocholithiasis.

**Video 1** Peroral cholangioscopy-guided transpapillary gallbladder irrigation and cholecystolithotomy in the treatment of acute cholecystitis and cholelithiasis.


First, a cholangioscope fitted with a tapered transparent cap was successfully cannulated into the common bile duct (CBD) under direct vision. Since the opening of the cystic duct was clearly visualized under cholangioscopy ( Fig. 2 ), the cholangioscope was squeezed into the cystic duct with the help of the soft transparent cap. With the guidance of the transparent cap, the cholangioscope straightened and expanded the curved cystic duct to allow smooth insertion into the gallbladder ( Fig. 3 ). With repeated irrigation and suction using the cholangioscope, the cavity of the gallbladder became clean and several gallstones were observed ( Fig. 4 ). The gallstones were captured by a slim basket and transferred to the CBD under cholangioscopy ( Fig. 5 ). With incision and expansion of the papillary orifice, the CBD stones and gallbladder origin stones were extracted with a regular ERCP basket.

**Fig. 2:**
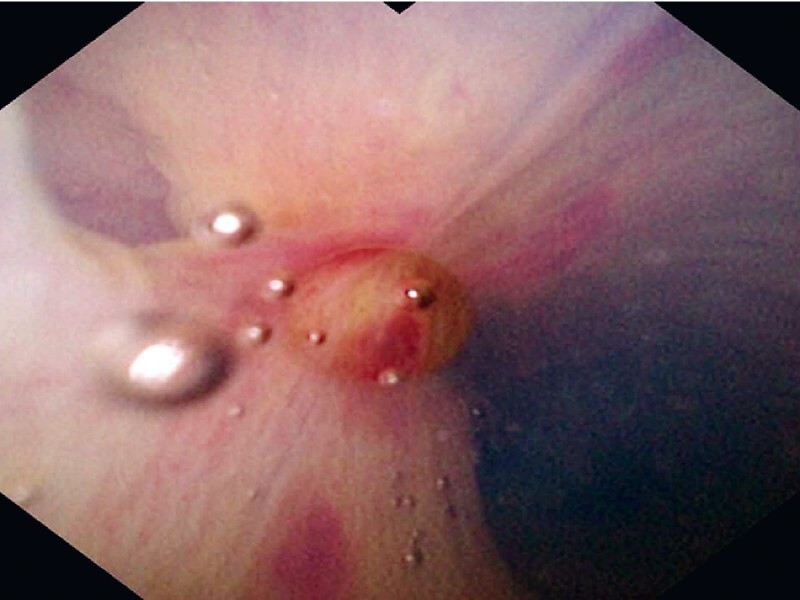
The opening of the cystic duct was directly observed under cholangioscopy.

**Fig. 3:**
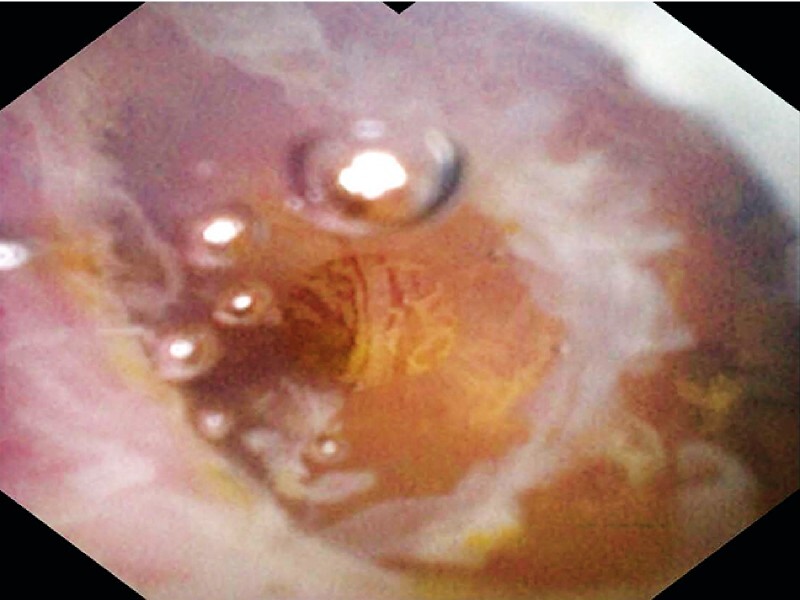
With the guidance of the transparent cap, the cholangioscope passed through the curved cystic duct.

**Fig. 4:**
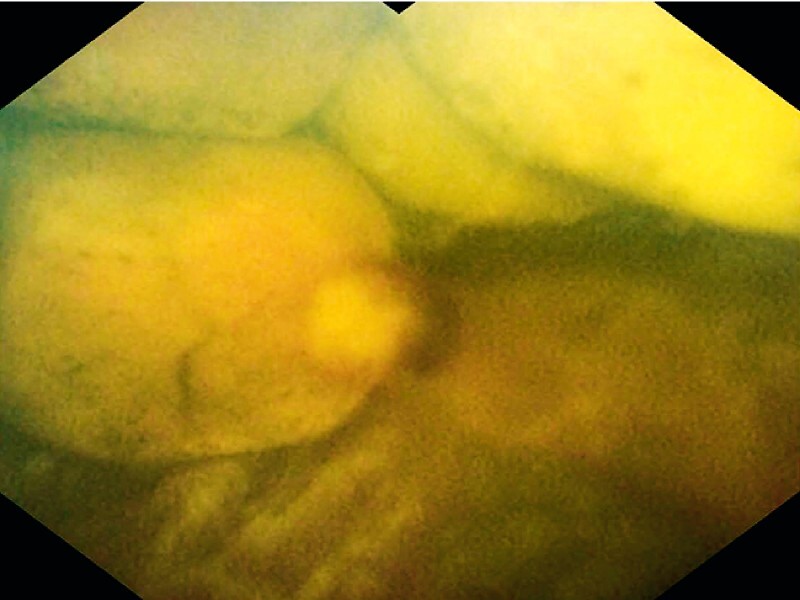
Sludge and stones were observed filling in the gallbladder.

**Fig. 5:**
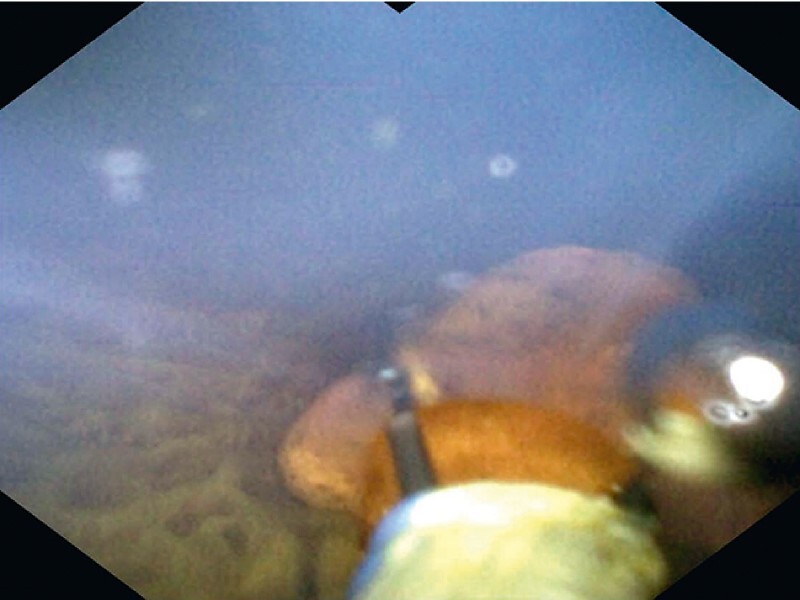
The gallstones were captured and extracted by a slim basket.

As one of the most effective treatments for acute cholecystitis, endoscopic transpapillary gallbladder drainage (ETGBD) is achieved completely through the natural pathway 1 . However, due to the tortuous anatomical structure of the gallbladder neck, the technical success rate is from 83 % to 88 % 2 , and some specific patients have inacceptable X-rays 3 4 , which limits the use of ETGBD. As we have established the cholangioscopy-guided biliary cannulating technique 5 , in this study we performed a gallbladder lavage and lithotomy using the transparent cap-covered cholangioscope. Since the cap-covered cholangioscope can effectively dilate and straighten the cystic duct, it has the potential to promote the success rate of ETGBD under direct vision.

Endoscopy_UCTN_Code_TTT_1AR_2AH

## Notes

Endoscopy E-Videos

https://eref.thieme.de/e-videos
*E-Videos* is an open access online section of the journal *Endoscopy* , reporting on interesting cases and new techniques in gastroenterological endoscopy. All papers include a high-quality video and are published with a Creative Commons CC-BY license. Endoscopy E-Videos qualify for HINARI discounts and waivers and eligibility is automatically checked during the submission process. We grant 100% waivers to articles whose corresponding authors are based in Group A countries and 50% waivers to those who are based in Group B countries as classified by Research4Life (see: https://www.research4life.org/access/eligibility/ ). This section has its own submission website at https://mc.manuscriptcentral.com/e-videos
